# The effect **of** laboratory critical value reporting **on** patient management **at** Siriraj Hospital – Thailand’s largest national tertiary referral center

**DOI:** 10.1371/journal.pone.0324594

**Published:** 2025-06-09

**Authors:** Preechaya Wongkrajang, Saharat Leelanuwatkul, Sairung Nuanin, Sathima Laiwejpithaya

**Affiliations:** Department of Clinical Pathology, Faculty of Medicine Siriraj Hospital, Mahidol University, Bangkok, Thailand; University of Health Sciences, Beyhekim Training and Research Hospital, TÜRKIYE

## Abstract

Critical laboratory values are life-threatening results that necessitate immediate medical intervention. Reporting these values according to established guidelines is essential for ensuring optimal patient safety and care quality. The aim of this study was to evaluate the laboratory critical value reporting system and the actions taken at Siriraj Hospital – Thailand’s oldest and largest teaching hospital – during January 2018. This study reviewed critical values from hematology, coagulation, and clinical chemistry tests over a one-month period. Patient management actions in response to critical values were classified into five categories: treatment, further investigation, monitoring, treatment combined with investigation, and other. Descriptive statistics were used to analyze the data in Microsoft Excel 2019, calculating the incidence of critical values, notification rates, and management actions. Of the 253,537 tests that were performed, 2,722 critical levels were found, indicating an incidence rate of 1.1%. Hemoglobin and potassium were the most frequently observed critical parameters, accounting for 25.61% and 23.99% of cases, respectively. The rate of notification varied depending on the specific parameter and patient category. For critical glucose and potassium levels, the most common response was close monitoring within 30 minutes, followed by treatment in 80% of cases. Hypermagnesemia, a condition linked to preeclampsia and treated with magnesium sulfate, required particularly careful monitoring. The 1.1% incidence of critical values in this study is high compared to previously published international data; however, this may be explained by the high volume of complex cases referred to our national tertiary referral center. Critical value reporting criteria should be established based on patient conditions and hospital management practices to reduce unnecessary alerts, optimize laboratory workload, and ensure high-quality patient care.

## Introduction

Critical laboratory values are defined as results that indicate life-threatening conditions that require immediate medical intervention [1]. Several key considerations are essential for the effective reporting of laboratory critical values, including the establishment of appropriate thresholds, timely notification, verification and documentation of the notification process (including read-back), identifying the responsible parties for both notifying and receiving the information, and determining the most effective communication method [2].

The recommended practices/guidelines surrounding laboratory critical value reporting are notably diverse. Professional organizations that publish standardized guidelines for the communication of laboratory critical values include the International Organization for Standardization (ISO) (i.e., ISO 15189:2022 [3], the College of American Pathologists (CAP) [4], and the Clinical and Laboratory Standards Institute (CLSI) [5]. These standardized guidelines outline critical elements of the notification process to ensure accuracy and efficiency in reporting. The essential elements of laboratory critical value reporting compared among 3 aforementioned guidelines are described in Table 1.

**Table 1:** Essential elements of laboratory critical value reporting compared among 3 different guidelines.

Standard	ISO 15189	CAP	CLSI
**Notification**	Mandatory	Mandatory	Mandatory
**Timing**	As soon as relevant based on the clinical information available	Immediate	1 hour
**To whom**	The user or other authorized person	A physician (or other clinical personnel responsible for the patient’s care)	Responsible caregivers
**By whom**	Not specified	Not specified	Lab director identifies suitable personnel (not the same person who performed the test
**Method**	Not specified	Verbal and non-verbal transmission	Verbal and non-verbal transmission
**Recording**	Date, time of acknowledgement; responsible person; person notified; results conveyed; verification of accuracy of communication; and, any difficulties encountered during notification	Date, time of acknowledgement; responsible laboratory; person notified; test results; read-back	Date, time of acknowledgement; patient identity; test name and result; operator identity of who has communicated the value; identity of recipient; read-back

**Abbreviations:** CAP, College of American Pathologists; CLSI, Clinical and Laboratory Standards Institute; ISO, International Organization for Standardization.

No previous study has investigated the laboratory critical value reporting system at a national tertiary care center in Thailand. Accordingly, the aim of this study was to evaluate the laboratory critical value reporting system and the resulting patient management actions taken at Siriraj Hospital during January 2018. Our investigation focused on laboratory critical value incidence rates, notification rates, notification-related efficiency, and the effect of these notifications on clinical decision-making. The results of this study will help to establish a valuable foundation for understanding laboratory critical value management in a Thai tertiary hospital context, to identify opportunities to enhance standards-based notification compliance, and to optimize patient care.

## Materials and methods

This study was conducted at the Department of Clinical Pathology, Faculty of Medicine Siriraj Hospital, Mahidol University, Bangkok, Thailand. A retrospective analysis of laboratory critical values from hematology, coagulation, and clinical chemistry tests that were performed during January 2018 was conducted. The data of critical value and patients’ management were accessed from 1 October 2018–31 May 2020. For personal identifiers, assigning pseudonyms were used instead of patient’s name and personal data. All raw data were stored in the principal investigator’s encrypted computer with password protection. The protocol for this study received ethical approval from the Siriraj Institutional Review Board (SIRB), Faculty of Medicine Siriraj Hospital, Mahidol University, Bangkok, Thailand (COA no. Si 547/2018). The authors were not required to obtain written informed consent from study subjects due to the retrospective, confidentiality-preserving design of this study.

In the absence of a standardized consensus on the lower limits and upper limits of critical value biomarkers from accreditation organizations, the Department of Clinical Pathology at our center establishes critical value thresholds via deliberation among clinical pathologists and clinicians every 1–2 years. The established lower and upper thresholds that were used at our center during the January 2018 study period are shown in Table 2.

**Table 2:** Lower and upper laboratory critical value limits.

Parameters	Lower critical value limit	Upper critical alue limit
**Chemistry**		
- Glucose (mg/dL)	<40	>400
- Potassium (mmol/L)	<2.9	>5.9
- Sodium (mmol/L)	<120	>160
- Ionized calcium (mg/dL)	<3	>6.4
- Magnesium (mg/dL)	<1	>4.7
**Arterial blood gas**		
- pH	<7.2	>7.6
- pCO_2_ (mmHg)	<20	>70
- pO_2_ (mmHg)	<40	None
**Hematology**		
- Activated partial thromboplastin time (APTT) (seconds)	None	>100
- International normalized ratio (INR)	None	>5
- Fibrinogen (mg/dL)	<100	None
- Hemoglobin (g/dL)	<7	None
- Platelet count (µL)	<10,000	>1,000,000
- White blood cell count (µL)	None	>100,000

**Abbreviations:** pCO_2_, partial pressure of carbon dioxide; pH, potential of hydrogen; pO_2_, partial pressure of oxygen.

The laboratory information system (LIS) in our department automatically alerts staff when a critical value is identified. The result is then verified to evaluate for any potential analytical errors. Once the accuracy of the result is confirmed, a nurse or other qualified healthcare provider in the relevant medical department is promptly notified of the laboratory critical value via telephone. The healthcare provider receiving the notification is required to confirm their identity and perform a readback of the value to minimize the risk of miscommunication. The laboratory technician then records the caregiver’s name and other relevant details in the LIS. The LIS maintains a detailed record of each critical value notification, including the date; the patient’s name, age, gender, ward, test parameter, and critical value; the name of the laboratory staff member responsible for communicating the result; the healthcare provider receiving the information; and, the time of notification. Notifications for critical glucose and potassium values are issued every time they occur, whereas critical values for other test results are only reported during the first occurrence of the critical value.

The actions analyzed in this study were categorized, as follows:

1. Treatment within 30 minutes
2. Further investigation within 30 minutes
3. Monitoring with no action taken within 30 minutes
4. Treatment plus further investigation within 30 minutes
5. Other – defined as 1) no action time was recorded, 2) the patient died, 3) the patient refused treatment, or 4) medical records were unavailable.

## Statistical analysis

All statistical analyses were performed using Microsoft Excel 2019 (Microsoft Corporation, Redmond, WA, USA). Demographic data and clinical data were analyzed using descriptive statistics. The percentage of critical values for each parameter was calculated using the following formulas: Percentage of Critical Values = (Number of Critical Values for the Parameter/ Total Number of Tests for that Parameter) × 100. The percentage of each parameter among all critical values was calculated using the formula: Percentage of All Critical Values = (Number of Critical Values for the Parameter/ Total Number of Critical Values) × 100.

The incidence of laboratory critical values for biochemical, arterial blood gas, and hematological parameters was calculated. That analysis included the total number of overall test results, the number of critical values, the percentage of critical values, and the critical value proportion. We also calculated the incidence of laboratory critical values for biochemical, arterial blood gas, and hematological parameters compared among inpatients (IPD), outpatients (OPD), emergency room (ER) cases, and all cases. That analysis included the number of critical values, the number of critical values notified, and the percentage of critical values that were notified. Lastly, we calculated the incidence of patient management actions recorded in the patient medical record after laboratory critical value result notification. That analysis included the number and percentage of patients received treatment, further investigation (FI), monitoring, treatment and further investigation, or other (defined as no action time recorded, the patient died, the patient rejected treatment, or there was no medical record) after laboratory critical result notification. Critical value data can be presented as a numerical value (count), percentage, a combination of both numbers and percentages, and/or as the mean ± standard deviation, where applicable.

## Results

During January 2018, a total of 253,537 test reports spanning 14 different test parameters were conducted at the Department of Clinical Pathology, Faculty of Medicine Siriraj Hospital, Mahidol University. Among those 253,537 tests, 2,722 (1.1%) were identified as critical values for an average of approximately 88 critical values per day. The majority of those critical values (1,813, 67%) were from inpatient department patients. Table 3 shows the incidence of laboratory critical values for biochemical, arterial blood gas, and hematological parameters, including the total number of overall test results, the number of critical values, the percentage of critical values, and the critical value proportion. Hemoglobin accounted for the highest percentage of all critical values (25.61%), followed by potassium (23.99%).

**Table 3:** Incidence of laboratory critical values for biochemical, arterial blood gas, and hematological parameters.

Parameters	Test results (n)	Critical values (n)	Percentage of critical values (%)	Percentage of all critical values^a^ (%)
**Chemistry**				
- Glucose	28,459	195	0.69	7.16
- Potassium	36,489	653	1.79	23.99
- Sodium	31,896	195	0.61	7.16
- Ionized calcium	3,020	22	0.73	0.81
- Magnesium	7,697	120	1.56	4.41
**Arterial blood gas**				
- Potential of hydrogen (pH)	2,429	99	4.08	3.64
- Partial pressure of carbon dioxide (pCO_2_)	2,429	122	5.02	4.48
- Partial pressure of oxygen (pO_2_)	2,429	219	9.02	8.05
**Hematology**				
- Activated partial thromboplastin time (APTT)	7,095	55	0.78	2.02
- International normalized ratio (INR)	9,415	102	1.08	3.75
- Fibrinogen	988	43	4.35	1.58
- Hemoglobin	40,397	697	1.73	25.61
- Platelet count	40,397	181	0.45	6.65
- White blood cell count	40,397	19	0.05	0.70

^a^Percentage of All Critical Values = (Number of Critical Values for the Parameter/ Total Number of Critical Values) × 100.

Table 4 shows the incidence of laboratory critical values for biochemical, arterial blood gas, and hematological parameters compared among inpatients, outpatients, emergency room patients, and all patients, including the number of critical values, the number of notifications, and the notification rates. For chemistry tests, the notification rates were generally between 80–90% across all patient groups, except for sodium, which had a lower notification rate of approximately 50%. Regarding arterial blood gas tests, the notification rates ranged from 70–100% for outpatients, but were lower (approximately 40%) for inpatients and among all patients. For hematology tests, activated partial thromboplastin time (APTT), international normalized ratio (INR), and fibrinogen were notified at rates ranging from 80% to 100%, whereas hemoglobin, platelet count, and white blood cell count notifications were lower ranging from 20% to 60% across all groups.

**Table 4:** Incidence of laboratory critical values compared among inpatients, outpatients, emergency room, and all patients.

Parameters	Inpatients			Outpatients			Emergency Room			All		
	Critical values (n)	Critical values notified (n)	% Critical values notification	Critical values (n)	Critical values notified (n)	% Critical values notification	Critical values (n)	Critical values notified (n)	% Critical values notification	Critical values (n)	Critical values notified (n)	% Critical values notification
**Chemistry**												
- Glucose	61	61	100.0	98	96	98.0	36	36	100.0	195	193	99.0
- Potassium	400	395	98.8	191	189	99.0	62	61	98.4	653	645	98.8
- Sodium	84	49	58.3	53	43	81.1	58	34	58.6	195	126	64.6
- Ionized calcium	22	18	81.8	0	0	0.0	0	0	0.0	22	18	81.8
- Magnesium	112	89	79.5	7	6	85.7	1	1	100.0	120	96	80.0
**Arterial blood gas**												
- pH	98	47	48.0	1	1	100.0	0	0	0.0	99	48	48.5
- pCO_2_	119	49	41.2	3	2	66.7	0	0	0.0	122	51	41.8
- pO_2_	219	87	39.7	0	0	0.0	0	0	0.0	219	87	39.7
**Hematology**												
- APTT	52	47	90.4	2	2	100.0	1	1	100.0	55	50	90.9
- INR	37	33	89.2	52	46	88.5	13	10	76.9	102	89	87.3
- Fibrinogen	40	32	80.0	2	2	100.0	1	1	100.0	43	35	81.4
- Hemoglobin	409	209	51.1	218	132	60.6	70	42	60.0	697	383	54.9
- Platelet count	145	32	22.1	25	6	24.0	11	6	54.5	181	44	24.3
- WBC count	15	3	20.0	3	2	66.7	1	0	0.0	19	5	26.3
**All**	**1,813**	**1,151**	**63.5**	**655**	**527**	**80.5**	**254**	**192**	**75.6**	**2,722**	**1,870**	**68.7**

**Abbreviations:** APTT, activated partial thromboplastin time; INR, international normalized ratio; n, number; pCO_2_, partial pressure of carbon dioxide; pH, potential of hydrogen; pO_2_, partial pressure of oxygen; WBC, white blood cell.

Table 5 describes the causes (prior critical value or autoverification) of unnotified laboratory critical values for biochemical, arterial blood gas, and hematological parameters. For sodium, the primary cause was autoverification (82.6%). In contrast, previously reported critical value or ‘prior critical value’ was the cause for the other parameters.

**Table 5:** Causes of unnotified laboratory critical values.

Parameters	Cause of unnotified critical value		
	Prior critical value	Autoverification	Total
	Numbers (%)	Numbers (%)	Numbers
**Chemistry**			
- Glucose	2 (100.0)	0 (0.0)	2
- Potassium	8 (100.0)	0 (0.0)	8
- Sodium	12 (17.4)	57 (82.6)	69
- Ionized calcium	3 (75.0)	1 (25.0)	4
- Magnesium	11 (45.8)	13 (54.2)	24
**Arterial blood gas**			
- Potential of hydrogen (pH)	51 (100.0)	0 (0.0)	51
- Partial pressure of carbon dioxide (pCO_2_)	67 (94.4)	4 (5.6)	71
- Partial pressure of oxygen (pO_2_)	130 (98.5)	2 (1.5)	132
**Hematology**			
- Activated partial thromboplastin time (APTT)	3 (60.0)	2 (40.0)	5
- International normalized ratio (INR)	10 (76.9)	3 (23.1)	13
- Fibrinogen	7 (87.5)	1 (12.5)	8
- Hemoglobin	286 (91.1)	28 (8.9)	314
- Platelet count	88 (64.2)	49 (35.8)	137
- White blood cell count	7 (50.0)	7 (50.0)	14
	**All unnotified critical value**		**852**

Table 6 presents the number, relative frequency, and mean of laboratory critical values across inpatients, outpatients, emergency room patients, and all patients. Hemoglobin levels below 7 g/dL accounted for the highest frequency of critical values across all patient groups, followed by potassium levels below 2.8 mmol/L. In contrast, critical values for low sodium (<120 mmol/L), high glucose (>400 mg/dL), and elevated INR (>5) were predominantly observed in outpatients and emergency room cases.

**Table 6:** The number, relative frequency, and mean of critical values compared among inpatients, outpatients, emergency room, and all patients.

Parameters	Critical value (Numbers)				Frequency (%)				Mean value (mean±SD)			
	IPD	OPD	ER	All	IPD	OPD	ER	All	IPD	OPD	ER	All
**Chemistry**												
- Glucose <40 mg/dL	56	14	7	77	3.09	2.14	2.76	2.83	30.28 ± 7.63	30.29 ± 7.09	23.14 ± 9.91	29.61 ± 7.93
- Glucose >400 mg/dL	5	84	29	118	0.28	12.82	11.42	4.34	489.60 ± 56.62	567.61 ± 164.15	646.59 ± 201.86	583.71 ± 174.70
- Potassium <2.8 mmol/L	278	91	27	396	15.33	13.89	10.63	14.55	2.53 ± 0.21	2.49 ± 0.25	2.57 ± 0.13	2.52 ± 0.22
- Potassium >6.0 mmol/L	122	100	35	257	6.73	15.27	13.78	9.44	6.51 ± 0.67	6.51 ± 0.64	6.47 ± 0.48	6.51 ± 0.64
- Sodium < 120 mmol/L	54	44	52	150	2.98	6.72	20.47	5.51	115.39 ± 3.72	114.45 ± 4.90	114.29 ± 4.16	114.73 ± 4.24
- Sodium >160 mmol/L	30	9	6	45	1.65	1.37	2.36	1.65	166.73 ± 4.01	164.56 ± 4.77	166.50 ± 4.76	166.27 ± 4.25
- Ionized calcium <3 mg/dL	19	0	0	19	1.05	0.00	0.00	0.70	2.62 ± 0.30	–	–	2.62 ± 0.30
- Ionized calcium >6.4 mg/dL	3	0	0	3	0.17	0.00	0.00	0.11	6.80 ± 0.35	–	–	6.80 ± 0.35
- Magnesium <1 mg/dL	16	7	1	24	0.88	1.07	0.39	0.88	0.79 ± 0.12	0.70 ± 0.14	0.70^a^	0.76 ± 0.13
- Magnesium >4.7 mg/dL	96	0	0	96	5.30	0.00	0.00	3.53	5.94 ± 1.23	–	–	5.94 ± 1.23
**Arterial blood gas**												
- pH < 7.2	82	1	0	83	4.52	0.15	0.00	3.05	7.10 ± 0.08	6.80^a^	–	7.10 ± 0.09
- pH > 7.6	16	0	0	16	0.88	0.00	0.00	0.59	7.64 ± 0.03	–	–	7.64 ± 0.03
- pCO_2_ < 20 mmHg	67	3	0	70	3.70	0.46	0.00	2.57	17.40 ± 2.07	14.03 ± 5.23	–	17.26 ± 2.32
- pCO_2_ > 70 mmHg	52	0	0	52	2.87	0.00	0.00	1.91	83.60 ± 12.13	–	–	83.60 ± 12.13
- pO_2_ < 40 mmHg	219	0	0	219	12.08	0.00	0.00	8.05	32.68 ± 4.60	–	–	32.68 ± 4.60
**Hematology**												
- APTT >100 sec	52	2	1	55	2.87	0.31	0.39	2.02	134.98 ± 29.07	143.45 ± 57.06	111.30^a^	134.85 ± 29.52
- International normalized ratio (INR) >5	37	52	13	102	2.04	7.94	5.12	3.75	7.12 ± 2.37	6.93 ± 2.08	6.98 ± 1.87	7.01 ± 2.14
- Fibrinogen <100 mg/dL	40	2	1	43	2.21	0.31	0.39	1.58	67.41 ± 24.32	80.75 ± 25.81	58.30^a^	67.82 ± 23.98
- Hemoglobin <7 g/dL	409	218	70	697	22.56	33.28	27.56	25.61	6.19 ± 0.70	5.96 ± 0.90	5.87 ± 0.93	6.09 ± 0.80
- Platelet count <10,000 µL	127	15	10	152	7.00	2.29	3.94	5.58	6.00 ± 2.36	5.47 ± 2.85	4.8 ± 2.66	5.87 ± 2.44
- Platelet count >1,000,000 µL	18	10	1	29	0.99	1.53	0.39	1.07	1169.94 ± 82.01	1162.90 ± 126.61	1052.00^a^	1163.45 ± 98.53
- White blood cell count >100,000 µL	15	3	1	19	0.83	0.46	0.39	0.70	141.43 ± 67.27	266.02 ± 171.23	143.98	161.23 ± 94.61
**Total**	**1,813**	**655**	**254**	**2,722**	**100**	**100**	**100**	**100**				

^a^SD cannot be determined.

**Abbreviations:** APTT, activated partial thromboplastin time; ER, emergency room; IPD, inpatient department; OPD, outpatient department; pCO_2_, partial pressure of carbon dioxide; pH, potential of hydrogen; pO_2_, partial pressure of oxygen; SD, standard deviation.

Table 7 outlines the patient management actions documented in medical records following laboratory critical value notifications. Within the first 30 minutes, monitoring was the most common management action across all critical value parameters. APTT had the highest percentage of cases that received treatment, underwent further investigation, or both (44%), followed by potassium (29.8%). In contrast, glucose was predominantly managed through monitoring (94.3%). Further breakdowns of patient management actions by patient category (inpatients, outpatients, and emergency room patients) are presented in the supplementary tables: S1, S2, and S3 Tables, respectively.

**Table 7:** Patient management actions after critical value result notification.

Parameters	Treatment n (%)	Further investigation n (%)	Monitor n (%)	Treatment and further investigation n (%)	Other^a^ n (%)
**Chemistry**					
- Glucose	2 (1.0)	0 (0.0)	182 (94.3)	1 (0.5)	8 (4.1)
- Potassium	111 (17.2)	45 (7.0)	409 (63.4)	36 (5.6)	44 (6.8)
- Sodium	12 (9.5)	2 (1.6)	101 (80.2)	1 (0.8)	10 (7.9)
- Ionized calcium	2 (11.1)	0 (0.0)	15 (83.3)	0 (0.0)	1 (5.6)
- Magnesium	12 (12.5)	1 (1.0)	83 (86.5)	0 (0.0)	0 (0.0)
**Arterial blood gas**					
- Potential of hydrogen (pH)	9 (18.8)	1 (2.1)	35 (72.9)	1 (2.1)	2 (4.2)
- Partial pressure of carbon dioxide (pCO_2_)	7 (13.7)	0 (0.0)	41 (80.4)	0 (0.0)	3 (5.9)
- Partial pressure of oxygen (pO_2_)	1 (1.1)	1 (1.1)	53 (60.9)	0 (0.0)	32 (36.8)
**Hematology**					
- Activated partial thromboplastin time (APTT)	16 (32.0)	5 (10.0)	27 (54.0)	1 (2.0)	1 (2.0)
- International normalized ratio (INR)	5 (5.6)	1 (1.1)	79 (88.8)	1 (1.1)	3 (3.4)
- Fibrinogen	6 (17.1)	1 (2.9)	28 (80.0)	0 (0.0)	0 (0.0)
- Hemoglobin	33 (8.6)	5 (1.3)	312 (81.5)	6 (1.6)	27 (7.0)
- Platelet count	2 (4.5)	0 (0.0)	38 (86.4)	0 (0.0)	4 (9.1)
- WBC count	1 (20.0)	0 (0.0)	3 (60.0)	0 (0.0)	1 (20.0)

^a^No action time recorded, the patient died, the patient rejected treatment, or there was no medical record.

**Abbreviations:** n, numbers.

## Discussion

This study provides an overview of the laboratory critical value reporting system and the post-notification patient management actions taken at our center during a one-month study period. A thorough review of the various operational aspects of critical value reporting was conducted based on data retrospectively collected from January 2018. Few studies in critical value reporting have been conducted in Thailand, and none of those studies evaluated the actions taken after the laboratory receives a critical value report, such as the notification process and subsequent patient management actions [6]. This is also the first study in Thailand to investigate and report on the laboratory critical value reporting system at a university hospital.

The list of critical values should be established using published national standards as a benchmark, with adjustments made by clinicians at each center as needed [7,8]. Some studies recommend that critical values be categorized by age group (adult and pediatric patients), ward type, and patient status [9,10]. Customizing critical value thresholds based on patient age and medical conditions ensures appropriate and efficient clinician notification, enhancing patient management [11,12]. Our laboratory established its critical value thresholds based on reviews conducted by clinical pathologists and clinicians.

The incidence of critical values in our laboratory was 1.1%, similar to 0.96% (Zhejiang University First Affiliated Hospital, China) [13] and 1.02% (Mustafa Kemal University, Turkey) [14]. However, it was relatively higher compared to 0.4% (University Hospital of Bellvitge, Spain) [15], 0.49% (Beijing Tsinghua Changgung Hospital, School of Clinical Medicine, Tsinghua University, China) [16], 0.50% (Sri Venkateswara Institute of Medical Sciences, India) [8], and 0.57% (Sun Yat-sen University Ophthalmic Center, China) [17]. The observed variation in critical value incidence may be attributed to differences in the list of critical values, in the cutoff thresholds, and in patient demographics [8]. Moreover, our center is a university-based national tertiary care facility that is referred patients with complicated conditions from smaller healthcare centers in Thailand.

The most prevalent critical values (lower or upper) observed in our study were for hemoglobin, which contrasts with other studies in which potassium, or sodium were reported to be the most common critical results [8,14,18]. This difference between and among studies may be attributed to the high prevalence of thalassemia in Thailand (approximately 1%) compared to the prevalence of thalassemia in the aforecited study populations [19]. Beta-thalassemia, which often requires blood transfusions, is most prevalent in the Mediterranean, the Middle East, and Southeast Asia [20].

The majority of critical results in our study came from the inpatient group. Both glucose and potassium were nearly always notified across all patient groups, including IPD, OPD, ER, and all patients. In contrast – for arterial blood gas tests, laboratory staff notified results for outpatients in 70–100% of cases, but for only approximately 40% of patients in the IPD and all patient groups. Notification rates for platelet count and white blood cell count were lower in IPD and all patient groups, with platelet count having the lowest notification rate in the OPD group. Previous study reported that glucose and phosphate had the highest notification rates in the IPD group, that potassium and calcium values were more frequently critical in the OPD group, and that platelet count was the least notified parameter in both groups [15].

Additionally, we observed that the notification rate for critical values in outpatients was the highest (80%), which contrasts with other studies that report lower notification rates in outpatient settings [15,21]. The most common cause of critical value non-notification for sodium was autoverification, whereas the most common cause of non-notification for all other parameters was ‘prior critical value’.

Critical hypoglycemia refers to abnormally low glucose levels, typically defined within the range of 40–60 mg/dL. In contrast, critical hyperglycemia indicates abnormally high glucose levels, with thresholds generally set between 350–500 mg/dL [22–24]. In this study, the frequency of critical hypoglycemia was notably high in IPD patients, which aligns with findings of Heller et al. who reported a high incidence of hypoglycemia in hospital settings due to insulin therapy. They emphasized the urgency of addressing low glucose levels in critically ill patients to prevent potential neurological consequences [25]. Regarding hyperglycemia, we found that OPD patients exhibited higher glucose levels, which is consistent with the findings of Yahaya et al. who noted an increase in extreme hyperglycemia in OPD settings, primarily due to poor diabetes management [26]. Hypokalemia (<2.8 mmol/L) and hyperkalemia (>6.0 mmol/L) are critical values that are frequently encountered in hospitalized patients. Our study revealed a high frequency of hypokalemia in IPD patients, and a high frequency of hyperkalemia in ER patients. This is consistent with findings of Bhargava et al. who reported hypokalemia to be most prevalent in hospitalized patients (particularly those receiving diuretics) [27], and hyperkalemia to be most prevalent in the ER, likely due to acute kidney injury or cardiac issues [28]. ER patients also showed a high frequency of hyponatremia (20.47%). Similarly, Lindner et al. reported a high prevalence of hyponatremia in ER patients, especially those receiving diuretics, those with heart failure, and those diagnosed with syndrome of inappropriate antidiuresis [29].

Hypomagnesemia and hypocalcemia (<3 mg/dL) were both commonly observed in IPD patients in the present study. Similarly, Philip et al. reported that disturbances in magnesium and calcium levels are common among intensive care unit patients, particularly in those with renal or respiratory failure [30]. Consistent with the findings of Aal-Hamad et al. [31], we observed a high frequency of hypermagnesemia in IPD patients, especially among pregnant women with preeclampsia due to the use of high-dose intravenous magnesium to prevent eclamptic seizures. The occurrence of potential of hydrogen (pH) <7.2 was more frequent in IPD patients, whereas the prevalence of pH > 7.6 was rare and in alignment with the existing literature. Foucher et al. reported severe acidosis (pH < 7.2) to be more prevalent in critically ill patients with respiratory failure, sepsis, or shock, which likely explains our observed higher incidence of pH < 7.2 in inpatients [32]. Additionally, our observed high frequency of both low pCO_2_ (<20 mmHg) and elevated pCO_2_ (>70 mmHg) in IPD patients is supported by the findings of Patel et al. who reported respiratory acidosis due to hypoventilation (high pCO_2_) to be a common complication in the intensive care unit [33].

In our study, hemoglobin levels <7 g/dL and platelet counts <10,000 µL were more common in IPD patients. This is consistent with the findings of previous studies that found hospitalized patients to be more likely to present with severe anemia or thrombocytopenia due to underlying chronic disease or bone marrow suppression [34,35]. Moreover, we found a high frequency of INR > 5 in OPD patients due to anticoagulant use. This INR critical value typically requires management to prevent bleeding complications, depending on the result and the patient’s symptoms [36].

More than 90% of critical glucose values were monitored in our study. However, a detailed analysis revealed that 146 of the 182 patients (80.2%) also received treatment, which can be further categorized into two groups: 1) treatment administered more than 30 minutes after notification (78/182, 42.9%), and 2) treatment based on point-of-care glucose results (68/182, 37.4%). This is consistent with clinical guidelines that recommend immediate intervention for glucose abnormalities to prevent adverse neurological effects [37]. Rapid intervention within one hour of identifying critical glucose values is associated with improved patient outcomes [38]. Regarding critical potassium values, over 60% of cases were monitored. However, we found that 328 of the 415 patients (79.0%) received treatment more than 30 minutes after notification. Notably, approximately 8% (33/415) of patients who did not receive treatment had chronic kidney disease. These patients likely had less severe hyperkalemia or were managed conservatively [39]. Approximately 90% of patients with hypermagnesemia were monitored, and further analysis revealed that 41.5% (44/106) of these cases were associated with preeclampsia. Magnesium sulfate is commonly administered to manage preeclampsia, resulting in elevated magnesium levels that necessitate intensive monitoring [40].

Based on these findings, the laboratory may need to adjust the criteria for reporting critical values to better reflect patient characteristics, particularly in tertiary care hospitals. For example, since patients in our context may tolerate lower hemoglobin levels without needing immediate action, the cut-off for reporting critical hemoglobin levels could be lowered from 7 g/dL. Similarly, the thresholds for critical magnesium levels could be adjusted based on patient location. In our obstetric facility, particularly in the labor ward, a higher threshold than 4.7 mg/dL may be more appropriate since patients with preeclampsia who receive magnesium sulfate therapy often have magnesium levels in the target range of 5–9 mg/dL (4–7 mEq/L) [41]. Another area for potential revision is the notification protocol for critical glucose values. Instead of alerting clinicians for every occurrence, notifications could be limited to the first critical value because the patient is likely already being treated and monitored with point-of-care testing. Revising these reporting criteria and notification protocols could help minimize unnecessary alerts, reduce the workload for laboratory staff, and improve the overall efficiency of critical value reporting. However, any changes should be made in close collaboration with clinicians to ensure they align with the best clinical practices.

## Strengths and limitations

This study has several strengths. First, this is the first comprehensive analysis of laboratory critical value reporting at a Thai university-based tertiary referral hospital. Second, unlike other studies that focus on critical value reporting only, this study also analyzes resulting patient management actions, which yields insights into the clinical decision-making process. Third, our study’s inclusion of data from diverse patient categories (inpatients, outpatients, emergency patients, and all patients) provides a comprehensive overview of laboratory critical value management across different care settings and overall.

The study also has some mentionable limitations. First, our data was collected from a single-center, so our findings may not be generalizable to other care setting, especially those with different patient demographics or critical value thresholds. Second, our study’s retrospective design renders it vulnerable to missing/incomplete data and certain potential biases. Third, the one-month data collection period may not capture seasonal trends or variations in critical value incidence. Fourth and last, our study did not evaluate the long-term impact of the interventions on patient outcomes. That acknowledged, our study set forth to describe the post-notification patient management actions, not to evaluate their efficacy.

Future studies could expand upon these findings by conducting similar studies across multiple hospitals in Thailand to generalize the findings and compare critical value practices across different healthcare settings. Moreover, we will analyze data over a longer time frame to capture seasonal trends and provide a more robust understanding of critical value incidence. Last, we will examine the long-term outcomes for patients after critical value intervention to assess the impact on morbidity and mortality.

## Conclusions

This study is the first in-depth analysis of the laboratory critical value reporting system and resulting patient management actions taken at a university-based tertiary care hospital in Thailand. The incidence of critical values was a relatively high 1.1% compared to international studies, which is likely due to the complex cases referred to our national tertiary referral center. Key findings showed prompt intervention for critical glucose values and monitored management for potassium, especially in chronic kidney disease patients. Hypermagnesemia was frequently associated with preeclampsia, which is consistent with the clinical practice of using magnesium sulfate in preeclampsia patients. The results of this study emphasize the need for context-specific laboratory critical value management, which varies by patient subgroup and condition. This study establishes a valuable foundation for understanding laboratory critical value management in a Thai tertiary hospital context, and the findings highlight areas that can be improved to optimize both patient safety and quality of care.

## Supporting information

S1 Table Patient management actions following critical value notification in inpatients. (DOCX)

S2 Table Patient management actions following critical value notification in outpatients. (DOCX)

S3 Table Patient management actions following critical value notification in emergency room. (DOCX)
