# Supplementary material for: The effect of laboratory critical value reporting on patient management at Siriraj Hospital – Thailand’s largest national tertiary referral center
Source: PLoS One. 2025 Jun 9;20(6):e0324594. doi: 10.1371/journal.pone.0324594 (PMC12148148; doi:10.1371/journal.pone.0324594)
Supplement: S1 Table — (DOCX) [file pone.0324594.s001.docx]

**S1 Table. Patient management actions following critical value notification in inpatients**

| **Parameters** | **Inpatients** | | | | |
| --- | --- | --- | --- | --- | --- |
|  | **Treatment**  **n (%)** | **Further investigation**  **n (%)** | **Monitor**  **n (%)** | **Treatment and further investigation**  **n (%)** | **Other**  **n (%)** |
| **Chemistry** |  |  |  |  |  |
| - Glucose | 0 (0.0) | 0 (0.0) | 59 (96.7) | 1 (1.6) | 1 (1.6) |
| - Potassium | 92 (23.3) | 17 (4.3) | 249 (63.0) | 23 (5.8) | 14 (3.5) |
| - Sodium | 6 (12.2) | 1 (2.0) | 42 (85.7) | 0 (0.0) | 0 (0.0) |
| - Ionized calcium | 2 (11.1) | 0 (0.0) | 15 (83.3) | 0 (0.0) | 1 (5.6) |
| - Magnesium | 11 (12.4) | 1 (1.1) | 77 (86.5) | 0 (0.0) | 0 (0.0) |
| **Arterial blood gas** |  |  |  |  |  |
| - Potential of hydrogen (pH) | 8 (17.0) | 1 (2.1) | 35 (74.5) | 1 (2.1) | 2 (4.3) |
| - Partial pressure of carbon dioxide (pCO_2_) | 6 (12.2) | 0 (0.0) | 40 (81.6) | 0 (0.0) | 3 (6.1) |
| - Partial pressure of oxygen (pO_2_) | 1 (1.1) | 1 (1.1) | 53 (60.9) | 0 (0.0) | 32 (36.8) |
| **Hematology** |  |  |  |  |  |
| - Activated partial thromboplastin time (APTT) | 16 (34.0) | 5 (10.6) | 24 (51.1) | 1 (2.1) | 1 (2.1) |
| - International normalized ratio (INR) | 3 (9.1) | 1 (3.0) | 27 (81.8) | 1 (3.0) | 1 (3.0) |
| - Fibrinogen | 5 (15.6) | 1 (3.1) | 26 (81.3) | 0 (0.0) | 0 (0.0) |
| - Hemoglobin | 21 (10.0) | 2 (1.0) | 177 (84.7) | 3 (1.4) | 6 (2.9) |
| - Platelet count | 2 (6.3) | 0 (0.0) | 30 (93.8) | 0 (0.0) | 0 (0.0) |
| - White blood cell count | 0 (0.0) | 0 (0.0) | 2 (66.7) | 0 (0.0) | 1 (33.3) |
