# Supplementary material for: The effect of laboratory critical value reporting on patient management at Siriraj Hospital – Thailand’s largest national tertiary referral center
Source: PLoS One. 2025 Jun 9;20(6):e0324594. doi: 10.1371/journal.pone.0324594 (PMC12148148; doi:10.1371/journal.pone.0324594)
Supplement: S2 Table — (DOCX) [file pone.0324594.s002.docx]

**S2 Table. Patient management actions following critical value notification in outpatients**

| **Parameters** | **Outpatients** | | | | |
| --- | --- | --- | --- | --- | --- |
|  | **Treatment**  **n (%)** | **Further investigation**  **n (%)** | **Monitor**  **n (%)** | **Treatment and further investigation**  **n (%)** | **Other**  **n (%)** |
| **Chemistry** |  |  |  |  |  |
| - Glucose | 2 (2.1) | 0 (0.0) | 88 (91.7) | 0 (0.0) | 6 (6.3) |
| - Potassium | 12 (6.3) | 20 (10.6) | 126 (66.7) | 5 (2.6) | 26 (13.8) |
| - Sodium | 4 (9.3) | 1 (2.3) | 31 (72.1) | 0 (0.0) | 7 (16.3) |
| - Ionized calcium | 0 (0.0) | 0 (0.0) | 0 (0.0) | 0 (0.0) | 0 (0.0) |
| - Magnesium | 1 (16.7) | 0 (0.0) | 5 (83.3) | 0 (0.0) | 0 (0.0) |
| **Arterial blood gas** |  |  |  |  |  |
| - Potential of hydrogen (pH) | 1 (100) | 0 (0.0) | 0 (0.0) | 0 (0.0) | 0 (0.0) |
| - Partial pressure of carbon dioxide (pCO_2_) | 1 (50.0) | 0 (0.0) | 1 (50.0) | 0 (0.0) | 0 (0.0) |
| - Partial pressure of oxygen (pO_2_) | 0 (0.0) | 0 (0.0) | 0 (0.0) | 0 (0.0) | 0 (0.0) |
| **Hematology** |  |  |  |  |  |
| - Activated partial thromboplastin time (APTT) | 0 (0.0) | 0 (0.0) | 2 (100) | 0 (0.0) | 0 (0.0) |
| - International normalized ratio (INR) | 1 (2.2) | 0 (0.0) | 43 (93.5) | 0 (0.0) | 2 (4.3) |
| - Fibrinogen | 0 (0.0) | 0 (0.0) | 2 (100) | 0 (0.0) | 0 (0.0) |
| - Hemoglobin | 9 (6.8) | 1 (0.8) | 103 (78.0) | 2 (1.5) | 17 (12.9) |
| - Platelet count | 0 (0.0) | 0 (0.0) | 4 (66.7) | 0 (0.0) | 2 (33.3) |
| - White blood cell count | 1 (50.0) | 0 (0.0) | 1 (50.0) | 0 (0.0) | 0 (0.0) |
