# Supplementary material for: The effect of laboratory critical value reporting on patient management at Siriraj Hospital – Thailand’s largest national tertiary referral center
Source: PLoS One. 2025 Jun 9;20(6):e0324594. doi: 10.1371/journal.pone.0324594 (PMC12148148; doi:10.1371/journal.pone.0324594)
Supplement: S3 Table — (DOCX) [file pone.0324594.s003.docx]

**S3 Table. Patient management actions following critical value notification in emergency room**

| **Parameters** | **ER** | | | | |
| --- | --- | --- | --- | --- | --- |
|  | **Treatment**  **n (%)** | **Further investigation**  **n (%)** | **Monitor**  **n (%)** | **Treatment and further investigation**  **n (%)** | **Other**  **n (%)** |
| **Chemistry** |  |  |  |  |  |
| - Glucose | 0 (0.0) | 0 (0.0) | 35 (97.2) | 0 (0.0) | 1 (2.8) |
| - Potassium | 7 (11.5) | 8 (13.1) | 34 (55.7) | 8 (13.1) | 4 (6.6) |
| - Sodium | 2 (5.9) | 0 (0.0) | 28 (82.4) | 1 (2.9) | 3 (8.8) |
| - Ionized calcium | 0 (0.0) | 0 (0.0) | 0 (0.0) | 0 (0.0) | 0 (0.0) |
| - Magnesium | 0 (0.0) | 0 (0.0) | 1 (100) | 0 (0.0) | 0 (0.0) |
| **Arterial blood gas** |  |  |  |  |  |
| - Potential of hydrogen (pH) | 0 (0.0) | 0 (0.0) | 0 (0.0) | 0 (0.0) | 0 (0.0) |
| - Partial pressure of carbon dioxide (pCO_2_) | 0 (0.0) | 0 (0.0) | 0 (0.0) | 0 (0.0) | 0 (0.0) |
| - Partial pressure of oxygen (pO_2_) | 0 (0.0) | 0 (0.0) | 0 (0.0) | 0 (0.0) | 0 (0.0) |
| **Hematology** |  |  |  |  |  |
| - Activated partial thromboplastin time (APTT) | 0 (0.0) | 0 (0.0) | 1 (100) | 0 (0.0) | 0 (0.0) |
| - International normalized ratio (INR) | 1 (10.0) | 0 (0.0) | 9 (90.0) | 0 (0.0) | 0 (0.0) |
| - Fibrinogen | 1 (100) | 0 (0.0) | 0 (0.0) | 0 (0.0) | 0 (0.0) |
| - Hemoglobin | 3 (7.1) | 2 (4.8) | 32 (76.2) | 1 (2.4) | 4 (9.5) |
| - Platelet count | 0 (0.0) | 0 (0.0) | 4 (66.7) | 0 (0.0) | 2 (33.3) |
| - White blood cell count | 0 (0.0) | 0 (0.0) | 0 (0.0) | 0 (0.0) | 0 (0.0) |
